# Facets of ICP-MS and their potential in the medical sciences—Part 2: nanomedicine, immunochemistry, mass cytometry, and bioassays

**DOI:** 10.1007/s00216-022-04260-8

**Published:** 2022-08-31

**Authors:** David Clases, Raquel Gonzalez de Vega

**Affiliations:** 1 Nano Mirco LAB, Institute of Chemistry, University of Graz, Graz, Austria; 2 TESLA-Analytical Chemistry, Institute of Chemistry, University of Graz, Graz, Austria

**Keywords:** Inductively coupled plasma–mass spectrometry, Mass cytometry, Nanotechnology, Single event ICP-MS, Bioassays, Nanomedicine

## Abstract

Inductively coupled–plasma mass spectrometry (ICP-MS) has transformed our knowledge on the role of trace and major elements in biology and has emerged as the most versatile technique in elemental mass spectrometry. The scope of ICP-MS has dramatically changed since its inception, and nowadays, it is a mature platform technology that is compatible with chromatographic and laser ablation (LA) systems. Over the last decades, it kept pace with various technological advances and was inspired by interdisciplinary approaches which endorsed new areas of applications. While the first part of this review was dedicated to fundamentals in ICP-MS, its hyphenated techniques and the application in biomonitoring, isotope ratio analysis, elemental speciation analysis, and elemental bioimaging, this second part will introduce relatively current directions in ICP-MS and their potential to provide novel perspectives in the medical sciences. In this context, current directions for the characterisation of novel nanomaterials which are considered for biomedical applications like drug delivery and imaging platforms will be discussed while considering different facets of ICP-MS including single event analysis and dedicated hyphenated techniques. Subsequently, immunochemistry techniques will be reviewed in their capability to expand the scope of ICP-MS enabling analysis of a large range of biomolecules alongside elements. These methods inspired mass cytometry and imaging mass cytometry and have the potential to transform diagnostics and treatment by offering new paradigms for personalised medicine. Finally, the interlacing of immunochemistry methods, single event analysis, and functional nanomaterials has opened new horizons to design novel bioassays which promise potential as assets for clinical applications and larger screening programs and will be discussed in their capabilities to detect low-level proteins and nucleic acids.

**Graphical abstract:**

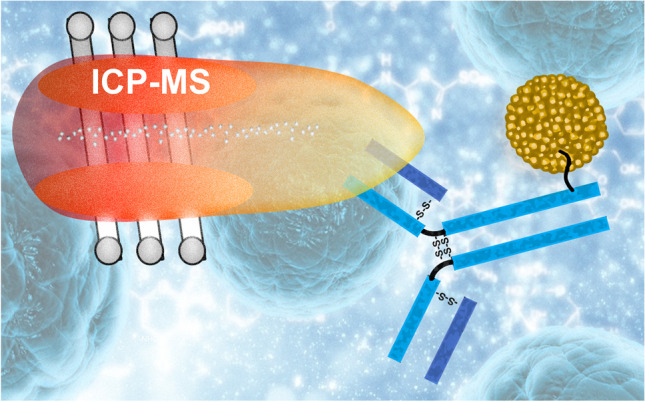

## Introduction

About 40 years after its commercial introduction, ICP-MS emerged as an extremely versatile technique with a vast potential in the medical sciences. The versatility of ICP-MS is anchored in its diverse facets which were promoted by recent technological innovations, the implementation of novel methodologies, and their interdisciplinary application. Some of these facets have been recognised in the medical research communities and efforts are directed to enable clinical translation. The associated applications and platforms have conspicuous opportunities to transform diagnostic and therapeutic paradigms, and to improve our understanding of biochemical pathways in normal physiology and diverse pathologies. Other facets of ICP-MS are less known and remain niche methods without current clinical relevance, but with potential to provide complementary points of views and alternative strategies to tackle current pressing biomedical research questions.

The first part of this review was dedicated to instrumentation, capabilities, and boundaries of ICP-MS, basic considerations for quantitative analyses, and applications relating to biomonitoring, elemental bioimaging, isotope ratios, and speciation analysis. These facets of ICP-MS built a fundament that has transformed our knowledge of elements and their biochemical and medical impact. However, further methodologies have been implemented over the past years and expanded the application range of ICP-MS. New paradigms and their biomedical implications will be discussed in this second part of this review. A focus will be directed to the capability of ICP-MS to analyse nanomaterials via single event analysis and hyphenated techniques. These facets will be discussed in their ability to provide additional information which are in high demand to develop and characterise novel nanomedicines considered for drug delivery and imaging platforms. Possibilities to expand the concepts of single event analysis to characterise individual cells will be discussed subsequently. Another focus will be directed to the integration of immunochemical methods into ICP-MS workflows. These methods endorsed the development of mass cytometry and imaging mass cytometry, which have a tremendous potential to provide new avenues for personalised medicine, diagnostics, and treatment in fields such as oncology. The interlacing of single event analysis, nanotechnology, and immunochemistry methods provided further options to develop dedicated bioassays offering new opportunities with promise for new inexpensive screening strategies.

## Nanomedicine

Nanotechnology is a driving innovative force in the medical sciences and offers new strategies for diagnostics and treatment. Nanomedical formulations may consist of polymers, peptides, liposomes, and inorganic material (e.g., metallic nanoparticles (NPs) and quantum dots (QDs)) and offer new opportunities for drug delivery platforms, diagnostic imaging, chemical sensing, and for targeted therapy. As for any drug, the development and approval process are rather inefficient, and consequently, nanomedicines passing the preclinical development have high failure rates in subsequent clinical studies. Previously, liposomal formulations and iron oxide-based NPs were successfully developed and approved [1], and a high annual number of new candidates are evaluated in clinical trials [2–4]. Given the potential and high utility of biomedical nanomaterials, preclinical research efforts are steadily increasing to develop new materials and to improve efficacy while limiting side effects. Different routes of administration are investigated, and oral, local, topical, and systemic approaches have previously been approved. However, given the possibility to access almost any part of the body, systemic approaches are favoured and have the most potential to impact clinical care [4–7].

The potential of NPs and their advantages over their bulk analogues in the medical realm is associated with the possibility to control electrical, optical, magnetic, and/or chemical properties via sizing, structuring, and functionalisation. A major bottleneck for the clinical translation of nanomedicine is related to analytical challenges for accurate and direct characterisation to recognise potentially adverse properties of candidates in an early stage of development. Techniques including fluorescence imaging, PET, CT imaging, electron microscopy, atomic force microscopy, dynamic light scattering, and nanoparticle tracking analysis are applicable to characterise NPs [8–10]. However, especially for the characterisation of inorganic particles like QDs and metallic nanomaterials, ICP-MS has capabilities that surpass other methods [10]. ICP-MS provides unique opportunities to detect and study particles regarding size and mass distributions, concentrations, stoichiometry, particle-particle and particle-matrix interactions, stability, dissolution, toxicity, biosafety, and fate. Besides the application of nanomaterials for drug delivery platforms and new imaging probes, bioassays profit from nanotechnological strategies which are especially interesting when paired with ICP-MS and reviewed in a later section of this review.

### Characterisation of nanomaterials in biological matrices

In its typical stand-alone configuration, ICP-MS has been an asset in combination with other analytical techniques to characterise emerging nanomaterials with potential to enhance imaging and drug delivery (e.g., QDs and up-conversion NPs [11]) by determining parameters relevant for pharmacological evaluation [12–14]. However, a more nano-dedicated facet of ICP-MS is its so-called single event mode. In this mode, dispersions are analysed at concentration levels where NPs enter the plasma individually. Consequently, particles are separately atomised and elemental ions are formed in isolated ion clouds, which may be extracted from the plasma for mass spectrometry. Each ion cloud is focussed into a discrete ion package, which generates a short pulse when hitting the detector. The number of pulses is proportional to the particle number concentration and the intensity of each pulse is proportional to the mass of the targeted isotopes in the detected particle. Simple calibration pathways translate signal histograms into models for mass and size distributions and analysis of the ionic background and counting statistics enable studies on particle stability by evaluation of aggregation and dissolution [15, 16]. Also known as single particle (SP) mode, this technique was first reported by Delgueldre et al. [17–19] and further improved by increasing ion transmission [15], operating faster mass analysers for the recording of individual ion clouds with several data points [20], and implementing technologies including the collision/reaction cell (CRC) [21], high-resolution MS [22], and tandem MS [23]. The application of sequential mass analysers precludes multi-elemental analysis in SP mode. The time which is required to scan and settle for the transmission of different m/z restricts the acquisition to only one m/z per particle. Current directions for faster data acquisition may increase the number of targetable isotopes per individual NP in the future [20]. Nonetheless at this stage, sequentially operating mass analysers are limited when targeting complex NPs consisting of various isotopes and elements. The simultaneous acquisition in ToF-based ICP-MS advanced the characterisation of complex nanomaterials significantly and it became a key technology to study particles consisting of more than one isotope and element. Fast integration times in SP ICP-MS, which ideally are within the microsecond range, produce large data sets which makes manual analysis unfeasible. Various vendors have introduced software packages to manage data acquisition, processing, calibration, and visualisation. However, also open-source software is available which improves comparison of data sets from different vendors, and offers more transparency and options for user-defined modifications [24]. Fundamentals, considerations, complementary techniques, and more information on single event analysis are available in reviews by Resano et al. and Meermann and Nischwitz [10, 25].

The high selectivity, sensitivity, and robustness of SP ICP-MS are well suited to trace nanomaterials in complex biological matrices and are useful to inquire important pharmacological parameters that are dependent on NP sizes, concentrations, surface modifications, dissolution and aggregation, and biochemical and cellular interactions [14, 16, 26]. For example, the dissolution of metallic particles may result into the formation of heavy metal ions which disrupt metabolic processes via irreversible inhibition of receptors and enzymes, and aggregation may lead to the precipitation of larger deposits which may trigger adverse physiological responses. Dissolution and aggregation of particles can be observed by monitoring the ionic background and by tracking particle sizes over time. However, also counting models are applicable to recognise aggregation [15, 27]. The uptake and adsorption of NPs on cells can be studied by sampling individual cells via single event analysis. Any NP associated with a cell is consequently analysed simultaneously and causes an integer multiple of the individual NP signal.

As the behaviour of particles is dependent on the biological matrix, previous simulations are vital to recognise and compensate potentially bio-incompatible properties of nanomedical candidates in an early stage. For example, physiological electrolyte concentrations may change the surface potential of particles and stimulate particle aggregation. Therefore, surface modification is often required to control aggregation and to ensure stability of particles. ICP-MS is applicable to study particle-particle and matrix-dependent effects as shown by Donahue et al., who studied the behaviour of PEGylated Au NPs at physiologically relevant saline conditions. SP ICP-MS was capable to follow aggregation at increasing salt levels, which could subsequently be prevented by increasing the PEG surface coverage [16]. Once NPs enter a biological system, their surfaces are immediately covered with abundant biomolecules. Especially for systemic applications, proteins were shown to form a corona which potentially changes physicochemical properties of particles [27, 28]. To investigate the NP-specific corona formation, separation techniques are often required before ICP-MS analysis. Fernandez-Iglesias and Bettmer investigated the protein corona built up on Au NPs in human serum using SEC-ICP-MS to separate and study corona associate proteins by analysing the sulphur integral to cysteine and methionine [28]. Interestingly, with increased NP diameter, the protein layers in the corona decreased. 10 nm sized particles showed 5–7 layers of proteins, whereas larger NPs (60 nm) had a protein monolayer. Similarly, Matczuk et al. employed CE as an alternative separation technique for online ICP-MS to characterise the protein corona [29]. The separation efficiency of SEC and other LC phases is linked to the surface area of the stationary phase as well as the pore sizes. As such, chromatographic resolution decreases for larger particles which require larger pore sizes for the size exclusion mechanism [30]. Therefore, SEC-ICP-MS is interesting for rather small nanomaterials including QDs [31–33]. CE-ICP-MS is gaining popularity for the analysis of nanomaterials as separation efficiency is not depending on a stationary phase and conditions can be manipulated easier to accommodate larger particles and to enable separations based on shape and surface charge [29, 34]. Franze et al. performed SP CE-ICP-MS and were able to separate different NPs while evaluating dispersions on a particle-to-particle basis to access particle number concentrations, size distributions, and elemental compositions [34]. Another interesting application of CE-ICP-MS was reported by Wroblewska et al., who studied the effects of NP surface modifications on the efficiency of drug binding for the application of nanocarriers [35]. Further information on the application of CE for the separation of metal containing NPs is available in a review by Aleksenko et al. [36]. The application of asymmetric flow field-flow fractionation (AF4) facilitates the separation of a wide range of particle sizes as shown for iron oxide NPs in serum. In the corresponding study, authors applied AF4-UV-MALS-ICP-MS/MS and SP ICP-MS to investigate matrix-dependent size modifications following spiking NPs into rat blood and plasma [37]. Similarly, Bocca et al. employed these methods to investigate Au and Ag NPs in human urine, blood, and serum. SP ICP-MS showed lower limits for the determination of particle number concentrations whereas AF4-ICP-MS enabled the analysis of smaller NPs [38].

As the manufacture and application of nanomaterials increase and while large amounts of nano contaminants are incidentally produced and discharged, exposure becomes inevitable and the requirement for biomonitoring and the evaluation of nano- and biosafety will certainly increase. The complementary application of SP ICP-MS and AF4-ICP-MS may be useful for biomonitoring to analyse NPs across large size and concentration scales. Within the environmental sciences, methods for the identification and characterisation of nanomaterials have been developed and may have a high utility for biomonitoring. Especially, ICP-ToF-MS [39] as well as non-target screening methods for sequential analysers [40] will become useful to identify and characterise a wide range of nano-scaled compounds regarding their impact on human health. This however will require dedicated sample preparation techniques and additional considerations. In a recent review, Laycock et al. pointed out various methods and protocols for the determination of nanomaterials in biological matrices via SP ICP-MS and the interested reader will find detailed information on strategies for sample collection, storage, preparation, and analysis [41].

### Imaging of nanomaterials in tissues

**Fig. 1:**
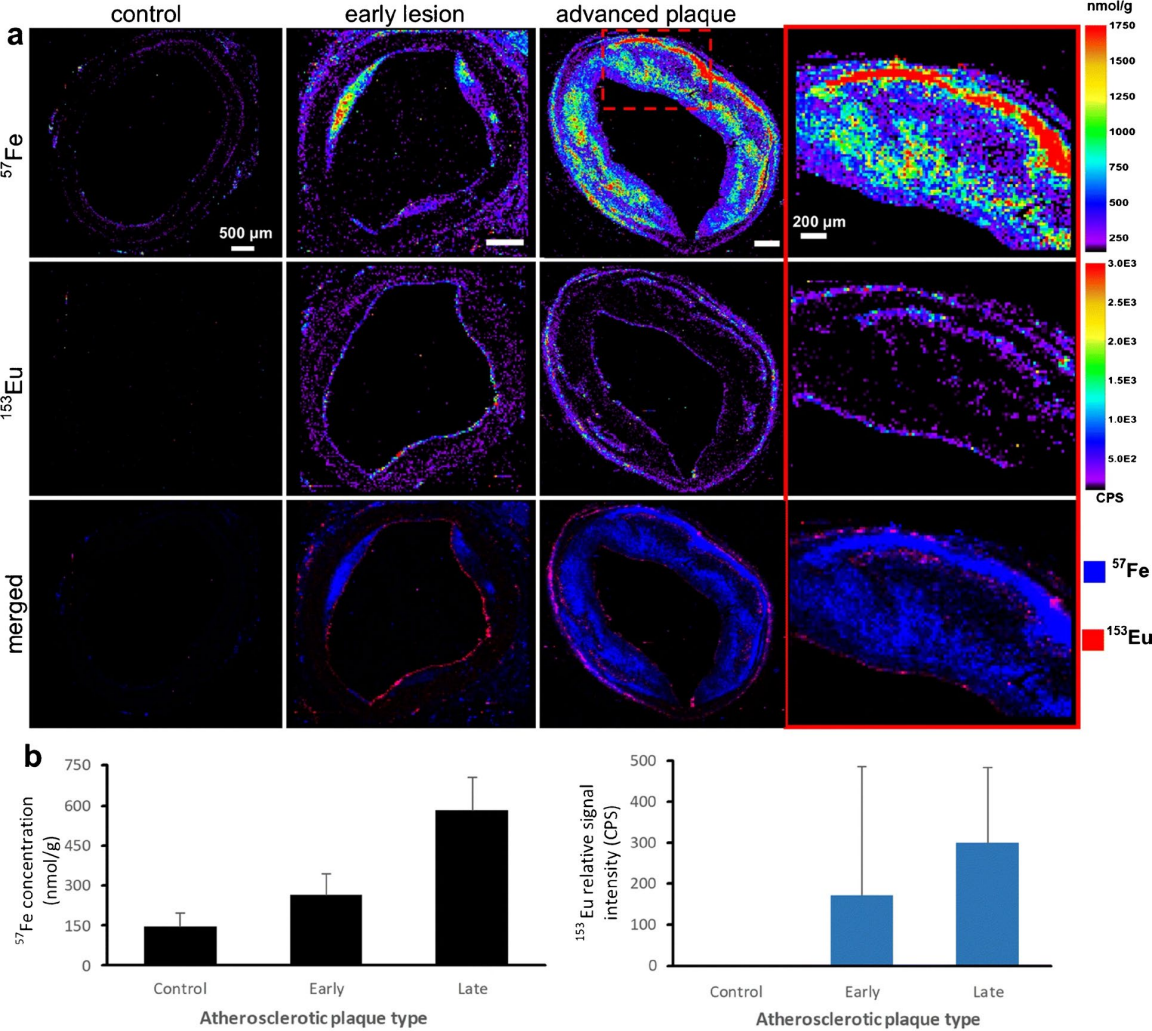
**a** Eu-doped iron oxide particles and a Gd-based contrast agent were analysed by LA-ICP-MS in control, early, and advanced atherosclerotic plaque progression. Scale bars, 500 µm, ROI, 200 µm. **b** Comparison of Fe concentrations and Eu signal intensity in atherosclerotic plaques. Reprinted from Springer Nature, Molecular Imaging and Biology, 23, 2021, 382–393, Microdistribution of magnetic resonance imaging contrast agents in atherosclerotic plaques determined by LA-ICP-MS and SR-μXRF imaging, Y.O. Uca, D. Hallmann, B. Hesse, C. Seim, N. Stolzenburg, H. Pietsch, J. Schnorr, M. Taupitz [46]. Copyright (2020), Uca et al.

While ICP-MS and SP ICP-MS as well as chromatography/CE-coupled ICP-MS provide options for the characterisation of biological interactions of nanomaterials, knowledge on the spatiotemporal distributions are of high value to shed light on cytotoxic mechanisms, translocations, retention, and fate of nanomedicine candidates. LA-ICP-MS was previously described (part 1) as a powerful tool to study the biological impact of toxicologically relevant metals and metalloids due to environmental exposure as well as to explore the role of endogenous elements in metabolic and pathologic pathways [42]. Therefore, it is not surprising that LA-ICP-MS is also suitable to study inorganic nanomaterials in biological systems. A variety of magnetic NPs are entering preclinical investigations and different formulations are considered to improve drug delivery and imaging and, especially, iron oxide NPs attract increasing interest [43, 44]. Scharlach et al. presented a LA-ICP-MS study quantifying iron oxide NPs in liver tissue sections and in atherosclerotic plaques. To distinguish endogenous and particulate Fe, particles were previously doped with Eu, which was targeted as proxy after administration [45]. Uca et al. used this method in combination with immunohistochemistry and synchrotron radiation µXRF spectroscopy to target Eu-doped iron oxide particles and Gd-based contrast agents used for MRI imaging. Similarly, they found distinct spatial distributions of iron oxide particles in atherosclerotic plaque walls (Figure 1), which indicated opportunities to use NPs to monitor plaque progression [46].

Au and Ag NPs have promise as drug delivery, photothermal, sensing, contrast, therapeutic, radio sensitising, and gene transfection agents [47]. Drescher et al. employed LA-ICP-MS to trace Au and Ag NPs in individual fibroblast cells following incubation experiments. They were able to reveal an accumulation of particles in the perinuclear region and developed a method to calibrate the number concentrations on a single cell level [48]. Büchner et al. applied LA-ICP-MS to study the Au NP uptake in 3T3 fibroblast cells. They combined their Au NP distribution data with SERS spectra to draw conclusion on the in vivo NP processing. In this complementary approach, they were also able to locate particles in perinuclear regions of cells, which were reorganised during mitosis, and further related NP uptake and localisation to molecular information of the particle’s corona [49]. The formation of the corona and biological interactions are highly depending on the surface functionalisation of particles. One example was provided by Mahmoud et al., who studied Au nanorods and investigated different surface ligands and surface charges regarding their impact on the interaction with skin and hair. They used LA-ICP-MS to investigate the penetration through different skin compartments [50].

Other types of nanomaterials considered for biomedical applications include polymer-based particles as well as QDs, which can be used as nanocarriers as well as for cell tracking, labelling, and imaging applications, respectively [51–53]. For example, Niehoff et al. employed poly(lactic-co-glycolic acid)-based nanocarriers containing a Pd-based photosensitiser. They employed LA-ICP-MS to follow the Pd signal and to study the accumulation of the drug in the outer layer of tumour spheroids. Compared to an administration without a NP-based carrier, significantly more homogeneous distributions of the photosensitiser were achieved [54]. The potential of QDs for in vivo applications is still under investigations and LA-ICP-MS is a useful tool to understand cytotoxicity and fate of QDs. For example, Wang et al. analysed the biodistribution of CdSe QDs following intravenous injection [55]. They were able to study fate, interactions, and biodistributions in a murine model and found that most QDs accumulated in the red pulps of the spleen, portal areas of the liver, and adrenal glands of the kidney within 1 h. They did not observe degradation but observed an immune response in the kidney. Pisonero et al. studied the QDs uptake and distribution in individual hippocampal neuronal cells and in human cervical carcinoma cells [56]. They resolved Cd distributions at cellular resolution and found QDs in the cytosol around the cell nucleus and further conducted single cell analysis to interpolate the number of QDs internalised per cell finding numbers between 3.5 ∙10^4^ and 48 ∙10^4^.

**Fig. 2:**
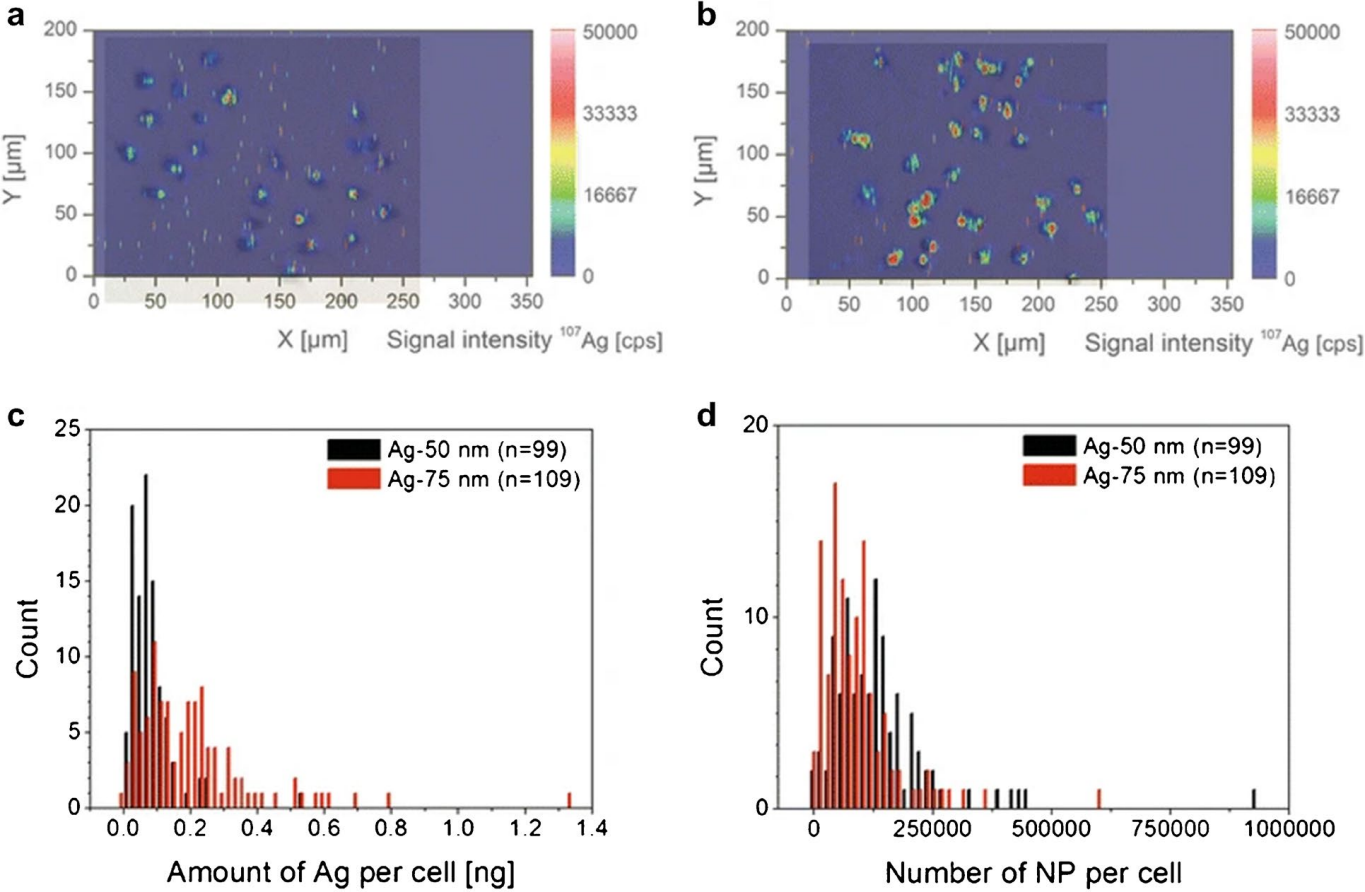
LA-ICP-MS was employed to study the uptake of differently sized Ag NPs (**a** 50 nm, **b** 75 nm). **c** and **d** show the histograms of the amount of Ag and Ag NP number in individual cells upon incubation. Reprinted from Springer Nature, Journal of Nanobiotechnology, 14, 2016, 50, Quantification and visualization of cellular uptake of TiO2 and Ag nanoparticles: comparison of different ICP-MS techniques, I-L. Hsiao, F.S. Bierkandt, P. Reichardt, A. Luch, Y-J. Huang, N. Jakubowski, J. Tentschert, A. Haase [57]. Copyright (2016), Hsiao et al.

A plethora of other nanomaterials are ubiquitous in everyday life and as exposure becomes inevitable, elemental imaging has potential to study the impact of emerging and little-known nano contaminants on physiology and to learn about uptake, effects, and fate. For example, Hsiao et al. applied LA-ICP-MS in addition to ICP-MS and SP ICP-MS to characterise and visualise the uptake of differently sized TiO_2_ and Ag NPs by neuroblastoma cells as shown in Figure 2. They found that smaller particles were taken up at higher rates and were also able to penetrate cell membranes. Larger particles were found to adsorb onto cells. Complementary SP ICP-MS of cell lysates indicated intracellular particle aggregation [57]. The translocation of NPs was investigated by Bishop et al. who performed quantitative imaging of Ag following intratracheal instillation of Ag NPs and reconstructed a three-dimensional representation of the Ag distribution in rat spleen [58]. Similarly, Reifschneider et al. studied the Ag NP uptake in alveolar macrophages, which were identified based on immunostaining of specific antigens [59]. Böhme et al. employed LA-ICP-MS, ICP-MS, and flow cytometry for studying the uptake of three differently sized Al_2_O_3_ NPs in human skin keratinocytes and lung epithelial [60]. Small model organisms are useful to interrogate the NP uptake due to environmental exposure. In a study by Zarco-Fernandez et al., zebra fish embryos were used as model organisms and exposed to Cd-based QDs and ionic Cd. Different bioconcentration factors indicated that the potential of QDs to bioaccumulate is lower than for ionic Cd [61].

A current development for the analysis of nanomaterials in the heterogeneous environment of biological tissues involves the combination of SP and LA-ICP-MS analysis to enable size-selective mapping. Li et al. tuned the laser fluence to disintegrate the biological matrix and to form an aerosol, while containing the integrity of Au NPs. They demonstrated the feasibility to image size distributions in tissues by analysing 60 and 80 nm Au NPs in matrix-matched kidney specimens and transferred their method to analyse 80 nm Au NPs in mouse liver [62]. This methodology was further advanced by Metarapi et al. by systematically optimising and simulating the influence of fluence, beam size and dwell time to minimise degradation, SP peak overlap, and interferences from ionic Au [63, 64]. In a subsequent study, they refined data processing to visualise size- and spatially resolved NP distributions [65]. The authors analysed plants following exposure experiments; however, as demonstrated by Li et al. [62] and as recently showcased by Nordhorn et al., who demonstrated the spatially and size-resolved Au NPs analysis in rat spleen after intratracheal instillation [66], methods can be applied in a biomedical framework.

## Immunochemistry-assisted elemental mass spectrometry

**Fig. 3:**
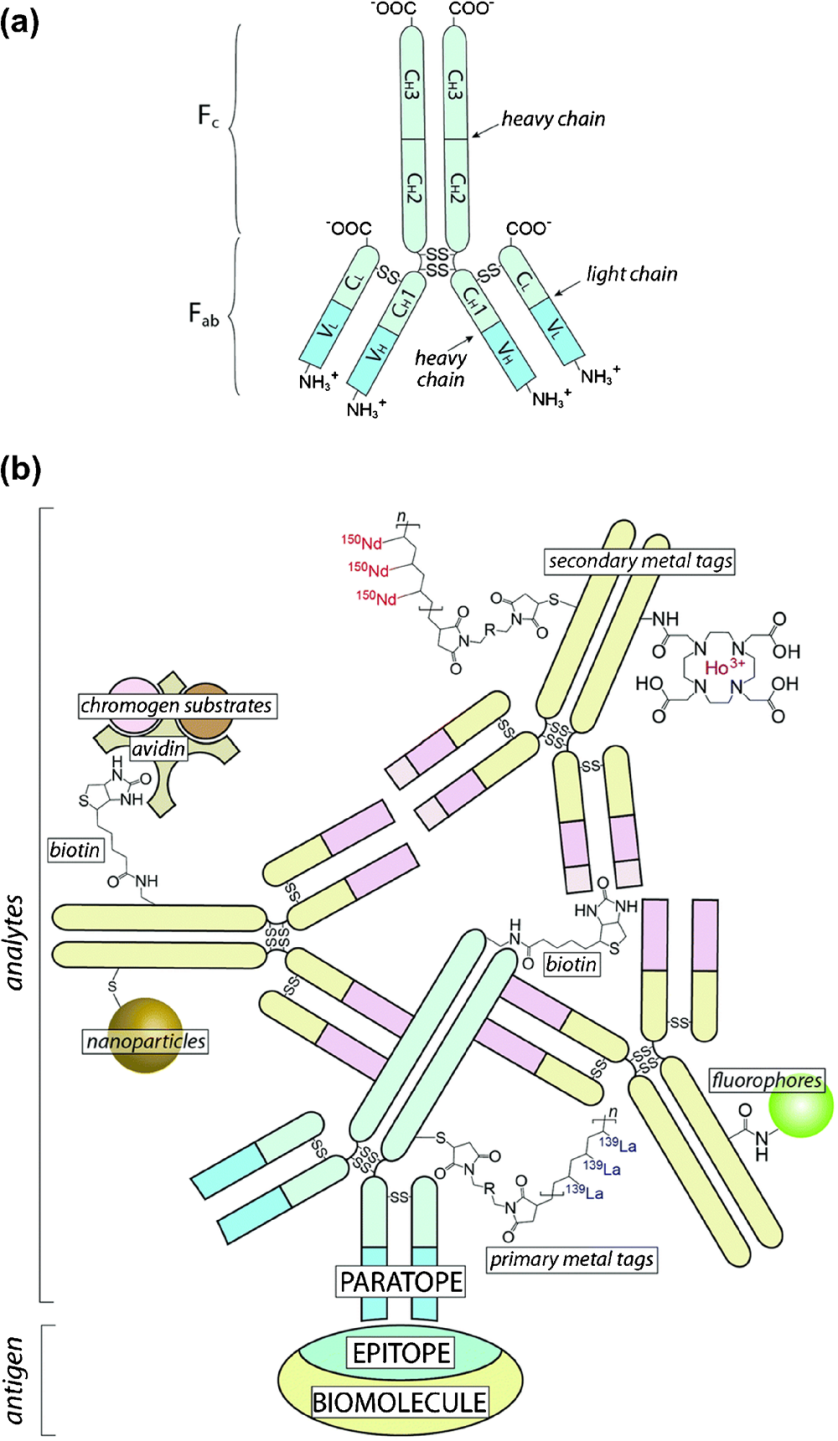
**a** A schematic illustration of an IgG antibody consisting of the F_c_ and F_ab_ regions, different subdomains, and a heavy and light chain. **b** shows the binding of a primary antibody to an epitope on a biomolecule. Secondary antibodies bind to its F_c_ region. Each antibody carries a certain label as reporter molecule. In case of LA-ICP-MS, metal labelling is required. Reproduced from Bishop et al. [67] with permission from the Royal Society of Chemistry

The immense biochemical diversity, the requirement to determine both levels and locations of biomolecules to interpret their biological impact, low concentrations, and the fact that targets must contain a compatible element set the boundaries for the application of ICP-MS techniques to locate and interpret biomolecules in a medical context. These boundaries were redefined by the implementation of immunochemical methods into ICP-MS-based workflows. In immunochemistry-assisted ICP-MS, metal-coded antibodies are exploited to target specific proteins, which become visible in ICP-MS by analysing the metal label as proxy. Especially in conjunction with hyphenated techniques and single event analysis, this provides entirely new opportunities to target previously inaccessible biochemical entities with high sensitivity and selectivity. The underlying principles and motivations of immunochemical methodologies go back to the necessity to interrogate tissue morphology and distribution of cell types to identify cellular structures for bioanalytical and diagnostic procedures. Histological stainings and dyes are routinely applied to mark biological substructures by harnessing chemical affinities. These methods are primarily qualitative and have limited specificity on a molecular level but may be improved by integrating antibodies as high affinity and highly selective probes [67]. In 1941, Coons et al. were the first to conjugate an antibody and a fluorescent label, which could be analysed optically as a proxy for the antibody and its target [68]. This technique was subsequently adopted for molecule-selective histological imaging via fluorescence microscopy and inspired new innovations providing an entirely new toolbox for selective chemical imaging of cells, whole organs, and single molecules [67]. For the application with ICP-MS, the labelling of a metal is required as proxy for the corresponding antibody, epitope, and biomolecule. Different labelling protocols are available to conjugate a metal tag to an antibody and different chemistries and commercial kits are applicable [67, 69] as shown in Figure 3. Currently, MAXPAR^®^ reagents are frequently used for antibody labelling and offer advantages by labelling a polymer with more than 100 binding sites for lanthanides. The polymer is conjugated to sulfhydryl groups from previously reduced disulphide bonds in the F_c_ regions of antibodies. The application of a polymer with some hundred lanthanide ions increases the labelling degree of antibodies and enhances sensitivity drastically. For characterisations and quantitative evaluations, exact labelling degrees can be determined by parallel SEC-ICP-MS to calibrate the average number of metal isotopes per antibody [70]. Typically, isotope enriched lanthanides are used to fill the binding sites of the polymers, which enhances sensitivity further. More importantly, however, a labelling strategy employing quantitatively enriched isotopes allows to associate one specific antibody with only one mass channel. The possibility to label panels of antibodies with more than 50 different isotopes pushes the limit of multiplexing to previously unseen levels. Current efforts to implement nanomaterials as isotopic labels may further have the potential to enhance labelling degrees and to promote highly sensitive analysis which is competitive with fluorescence-based analogues. Finally, besides antibodies, nucleotides can be labelled and utilised as probes to target specific DNA and RNA sequences and fragments. Alternatively, they may be applied as labelled aptamer, which exhibits high affinity to selectable epitopes [71].

Altogether, immunochemistry increased the reach of elemental mass spectrometry and shifted the scope of ICP-MS which now may operate more interdisciplinary at the interface of Metallomics, Genomics, and Proteomics. These different facets and their abilities in bioassays, imaging, and single cell analysis will be further discussed in the following sections.

## Single cell analysis

### Single cell analysis and mass cytometry

**Fig. 4:**
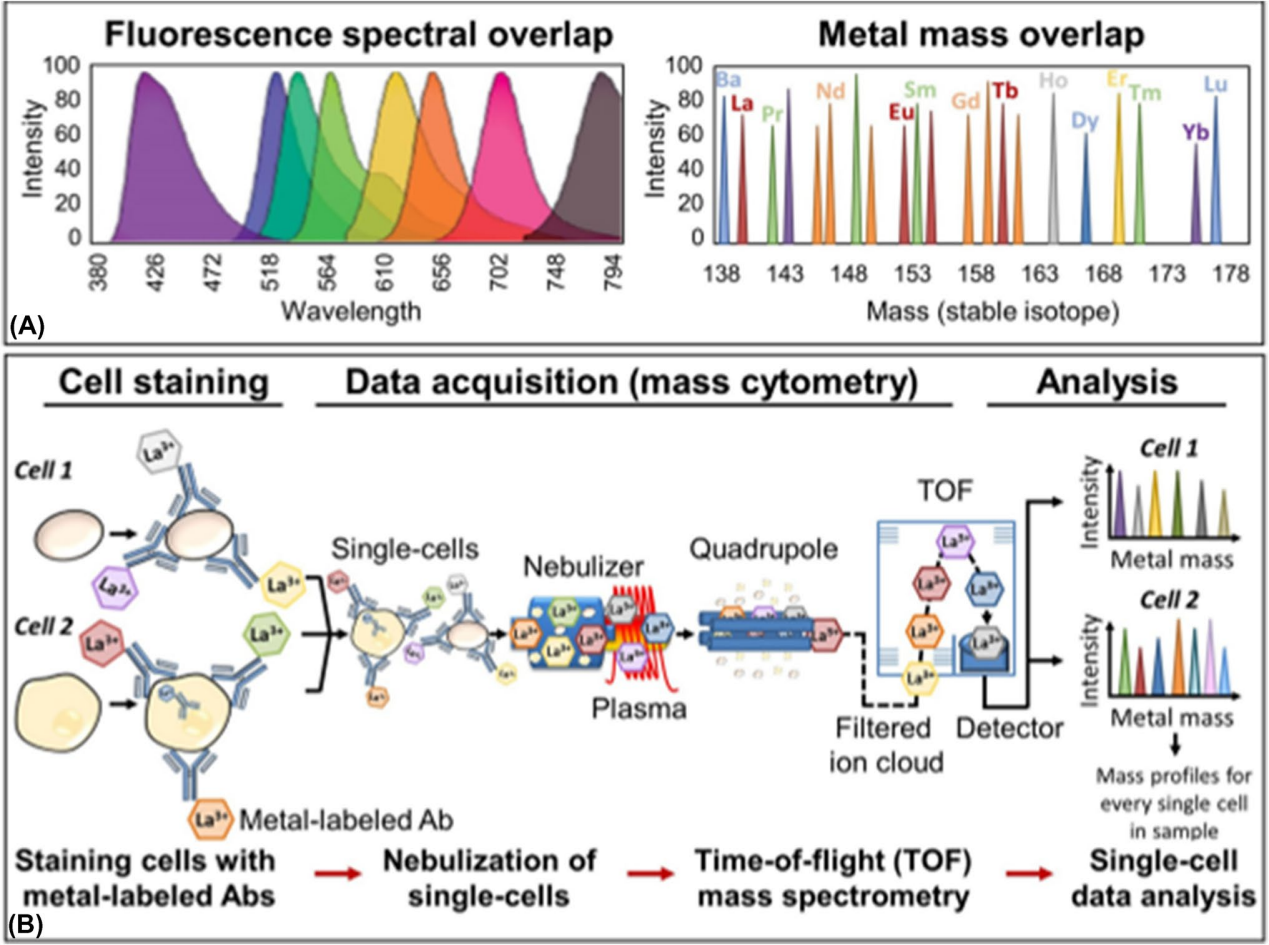
**A** compares the spectral overlap of fluorescent and metal labels. Unit mass resolution enables the simultaneous analysis of more than 50 isotopes, while fluorescence-based multiplexing is significantly limited by spectral overlap of emission bands. **B** shows the workflow of mass cytometry. Cells are incubated with metal-coded antibodies, which target specific epitopes on cells. SC ICP-MS targets metal labels as proxy for certain biomolecules and cells enabling the identification of different subsets. Reprinted from Platelets, Fourth Edition, T. A. Blair, A. L. Frelinger, A. D. Michelson, 35 – Flow Cytometry, 627–651. Copyright (2019), with permission from Elsevier [77]

The counting, identification, and further characterisation of individual cells has long been established in the medical sciences. While initially, cells were counted visually using a haemocytometer, today, flow cytometry is commonly employed to characterise individual cells based on optical effects like scattering and fluorescence [72]. The latter can be exploited by employing high affinity probes like antibodies or aptamers, which are labelled with a fluorescent reporting molecule. The fluorescence signal is theoretically proportional to the number of labels and consequently allows to draw conclusions about the quantity of targeted antigens. However, photobleaching, matrix effects, unspecific binding, and spectral overlaps of relatively broad emission bands (compare Figure 4A) often interfere and prevent quantification efforts. The overlap of emission bands further stipulates the maximum multiplexing degree in fluorescence-based IHC. Consequently, information on cell antigens, cell identities, and substructures are restricted to a few biochemical entities, which is a significant limitation in view of the vast biochemical and cellular diversity. However, these issues can be overcome by employing immunochemistry methods in conjunction with single event ICP-MS which adopts the principles of flow cytometry when analysing mass-coded antibodies. As pointed out in the previous paragraphs, SP ICP-MS is capable to enquire discrete structures on the nanoscale. However, besides nanostructures, also larger particles with dimensions on the micro-scale [73] as well as unicellular organisms can be analysed separately for elemental analysis [74, 75]. This fusion of flow cytometry and ICP-MS is known as mass cytometry and combines the strengths of both techniques by offering access to individual cells as well as isotopic information across a large dynamic range. The principles and workflow of mass cytometry is illustrated in Figure 4B. It is noteworthy that also some limitations are inherent upon fusion of both techniques. First, the cell throughput of ICP-MS is lower, especially when high transport efficiencies are required. Second, mass cytometry is a destructive technique and cells cannot be recovered for subsequent or repeated analysis. Third, compared against quantum efficient fluorophores, analysing isotope labels may provide less sensitivity complicating the analysis of low-level molecular entities. To increase sensitivity in mass cytometry, polymer tags containing binding sides for some hundred isotopes or nanoparticulate isotope labels may increase sensitivity competitively [76].

Access to a large range of antibodies, the commercial availability of prelabelled antibodies, and the option to perform labelling and characterisation on-site makes mass cytometry a versatile technique that may be employed for cell profiling and to explore therapeutic avenues. The rapid and high-dimensional analysis of cells promotes mass cytometry to identify major and rare cell subsets with unique biological activity and enables the establishment of models on cell variances and cellular responses. The sampling frequency of up to 1000 cells per second allows to pinpoint rare cell populations with essential biological functions, which would otherwise be overlooked. Also, protein levels, posttranslational modifications, and proteolysis can be investigated in a single experiment [76, 78]. However, the vast amount of information produced during a single experiment demands the integration of automated workflows and statistical tools to process and interpret the multiparametric data sets.

As noted by Behbehani, clinical implementation of mass cytometry appears extremely rewarding and powerful to advance personalised medicine via possibilities to identify cells and to complement the broad scope of genomic assays [79]. Besides the detection of rare cell populations, multiparametric analyses have also therapeutic value to identify cell-specific properties such as up-regulated signalling pathways, expression of immune checkpoint inhibits, or altered DNA damage response pathways [79]. The fact that mass cytometry is capable not only to pinpoint and characterise rare cell populations but also to explore therapeutic responses, endorses applications for example in the field of malignant and autoimmune diseases [80, 81]. The interested reader will find further information on the clinical potential, applications but also on the instrumentation, principles, methods, and data analysis in dedicated reviews [76, 78–80, 82, 83].

In the past decade, mass cytometry has evolved from an emerging technology to an accepted platform for high-dimensional single cell analysis and was mainly driven by the possibility to achieve higher multiplexing degrees [76]. Currently, more than 50 distinguishable isotopic labels enable simultaneous analysis of large biomolecule panels in individual cells. Due to the short signal event duration in single cell analysis and the requirement for multi-isotopic analyses on an individual cell, sequentially operating mass analysers are not suited and the ToF analyser is the only viable option to detect extended panels. The adoption of ToF technology led to a paradigm shift and improved the characterisation of complex cellular samples significantly. For medical applications, the commercial CyTOF platform was most frequently employed and attenuates low mass isotopes (< 80 amu) to focus on the high mass range which is reserved for isotope labels [84]. This strategy has advantages regarding spectral interferences and to increase the duty cycle but precludes the analysis of endogenous elements such as transition metals. However, it is noteworthy that endogenous elements are receiving increasing attention in single cell analysis and complementary data may be provided by interrogating fingerprints of major and trace elements to decipher cell types and pathologies. The option to target the entire mass range was provided by two other commercial platforms which employ notch filters and/or CRCs to mitigate interferences [85–88].

**Fig. 5:**
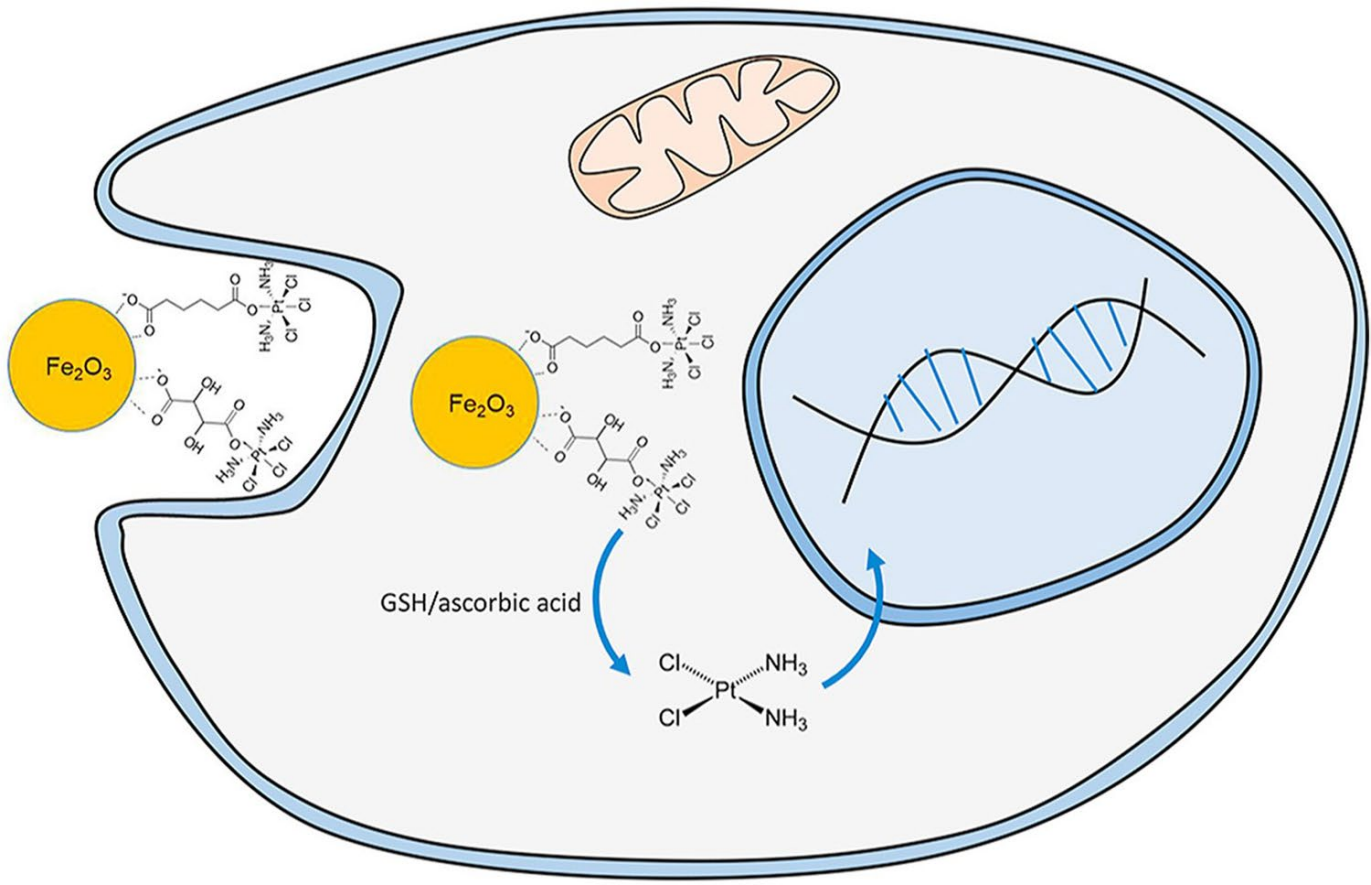
Schematic of the cell-nanodrug interaction. The cisplatin prodrug was conjugated to the surface of an iron oxide nanocarrier, which facilitated the transport across the cell membrane and increased DNA platination significantly. Reprinted from Analytica Chimica Acta, 1159, D. Turiel-Fernandez, L. Gutierrez-Romero, M. Corte-Rodriguez, J. Bettmer, M. Montes-Bayon, Ultrasmall iron oxide nanoparticles cisplatin (IV) prodrug nanoconjugate: ICP-MS based strategies to evaluate the formation and drug delivery capabilities in single cells, 338356 [44]. Copyright (2021), with permission from Elsevier

Efforts to analyse endogenous elements in individual cells have been increased in the last decade to associate cellular responses with environmental exposure, pathologies, and treatment. The methodical approach is concordant with mass cytometry with the difference that the lower mass range is investigated and that alternative mass analysers are frequently applied. The concept of single cell (SC) ICP-MS may further be expanded to include a variety of unicellular organisms including human and mammalian cell lines [26], bacteria [89], yeast [90], or plants [73]. Exemplarily studies on elemental exposure on a single cell level investigated the bioavailability of environmentally/toxicologically relevant element species (e.g., As- and Cr-based species) and aimed to interrogate cellular metabolism [91–93]. However, SC analysis enables further profiling of endogenous elements and the detection of disease-mediated up- and downregulations, which may have diagnostic utility to monitor cellular variations due to aberrant metabolic pathways. For example, Wang et al. found large variations of Fe, Cu, Mn, Zn, P, and S when comparing cancer and control cell lines [94]. Another current direction in SC ICP-MS is its application to investigate the cellular uptake of metallodrugs and nanomedical formulations. Studies on the uptake of metallodrugs are mainly focussed on anticancer agents with an emphasis on Pt-based agents [95, 96]. However, also Bi- [97], Gd- [96], Co- [98], and As-based drugs [99] were analysed regarding cellular accumulation. However, the SC ICP-MS workflow has value as a platform for toxicological and viability tests to study, promote, exclude, or modify novel therapeutic nano-candidates in an early stage of drug development. Using a SC approach, it is possible to study the internalisation of NPs as shown by Wei et al., who studied the uptake of Au NPs in MCF-7 cells and determined between 130 and 584 NPs per cell [100]. Another interesting example illustrated in Figure 5 was reported by Turiel-Fernandez et al., who employed SC ICP-MS to study an iron oxide-based anticancer drug delivery platform. A Pt-based prodrug was conjugated to the surface of particles and was released upon cellular internalisation for DNA platination. The use of iron oxide NPs with an average diameter of 6.6 nm promoted drug internalisation and 5-fold increased Pt levels were found relative to a cisplatin comparison [44]. The possibility to target metal-based drugs in different cells is further interesting to inquire cellular responses. It offers the fundament for models to investigate drug associated sensitivity and resilience on a cell-specific level. Further information on techniques in SC ICP-MS including elemental bioimaging of individual cells, instrumental considerations, calibration approaches, and further application may be found in recent reviews by Theiner et al. [101] and Corte-Rodriguez et al. [75].

Given that ToF technology may simultaneously analyse isotopes across the entire mass range, it seems plausible that future mass cytometry/SC ICP-MS approaches increasingly consider endogenous elements and/or metallodrugs next to isotopic immunolabels to identify drug responses in specific cell subsets as well as to complement and expand the panel of analytes.

### Protein bioimaging and imaging mass cytometry

The integration of immunochemical protocols for ICP-MS initiated a paradigm shift by making vast and previously foreign types of analytes accessible. Besides the application of single event analysis, immunochemistry methods may be used in hyphenated techniques like LC-ICP-MS and LA-ICP-MS to trace biomolecules. Especially LA-ICP-MS is interesting for the visualisation of biodistributions of molecules among endogenous elements and promotes a more interdisciplinary and holistic application of ICP-MS by consideration of both the Proteome and the Metallome [42]. Biomolecules such as membrane proteins, intercellular molecules associated with signalling, and intracellular targets including histones or DNA but also extracellular molecules like therapeutics can be analysed by exploiting isotopes of metals and metalloids as the common currency in ICP-MS [83]. This enables visualisation of the architecture, microenvironment, and morphology of tissues and may portrait pathologies from different angles by targeting novel types of relevant bio-indicators.

The application of metal-coded antibodies for imaging requires histological methods (immunohistochemistry (IHC)) for the incubation of the labelled antibody and the tissue. Tissues can generally be obtained as fresh-frozen, formalin-fixed, and paraffin-embedded [102]; however, antigen retrieval strategies may be required depending on the sample type and targeted antigen. The IHC method requires careful optimisation to avoid over- and under-staining and titrations as well as blocking procedures are commonly applied to maximise contrast and limit unspecific staining. Either primary or secondary antibodies may be labelled and applied for biomolecule imaging. The latter one is sometimes preferred as secondary antibodies are more affordable and may be repurposed for different experiments. However, due to the limited number of animal host species as source for secondary antibodies, higher multiplexing degrees can be obtained via labelling and application of primary antibodies [42].

**Fig. 6:**
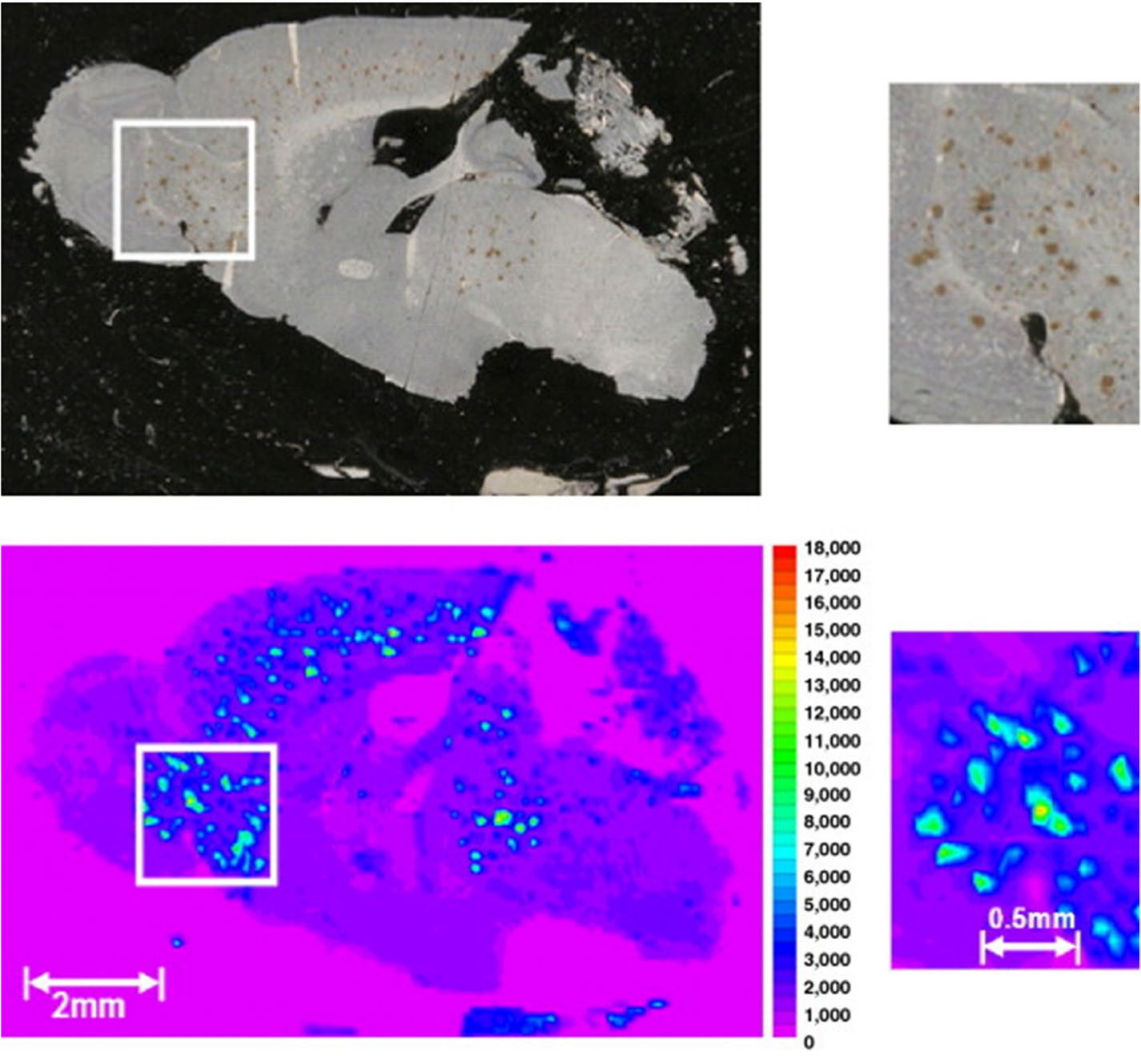
Image of a TASTPM mouse brain section, which was treated with a monoclonal antibody to reveal the β-amyloid protein distribution using nickel–DAB (top). LA-ICP-MS was used to interrogate the Ni distribution (bottom). Reprinted from Analytical Biochemistry, 346, R.W. Hutchinson, A.G. Cox, C.W. McLeod, P.S. Marshall, A. Harper, E.L. Dawson, D.R. Howlett, Imaging and spatial distribution of β-amyloid peptide and metal ions in Alzheimer’s plaques by laser ablation–inductively coupled plasma–mass spectrometry, 225–233 [103]. Copyright (2005), with permission from Elsevier

The first use of IHC and LA-ICP-MS for biomolecules imaging goes back to Hutchinson et al. (Figure 6) who resolved the distribution of β-amyloid in Alzheimer’s plaques in 2005 and Seuma et al. followed in 2008 with a multiplexing approach for the joint analysis of MUC-1 and HER-2 in breast cancer tissue utilising Au and Ag NPs as element labels [103, 104]. The multiplexing degree was gradually improved and besides metallic NPs and lanthanide labels, other isotope labels (e.g., I [105] or QDs [51]) and different labelling chemistries with distinct linker molecules (e.g., SCN-DOTA [106], MeCAT [107], and MAXPAR^®^ [108, 109]) were suggested for the marking of proteins and antibodies (compare Figure 3). Examples for multiplexed approaches include reports from Giesen et al., who analysed three tumour markers (HER-2, CK7, MUC-1) in breast cancer tissues [110], and Lores-Padin et al. reported the application of metal-based nanoparticles (Au, Ag, Pt) for the analysis of three proteins (MT1/2, CFH, and APP) associated with Alzheimer’s disease in eye sections [111]. Neumann et al. investigated a 7-membered antibody panel in a murine parkinsonian model [112], and Aljakna et al. performed a multiplexed analysis of 7 proteins in a human myocardial infarction model [113].

**Fig. 7:**
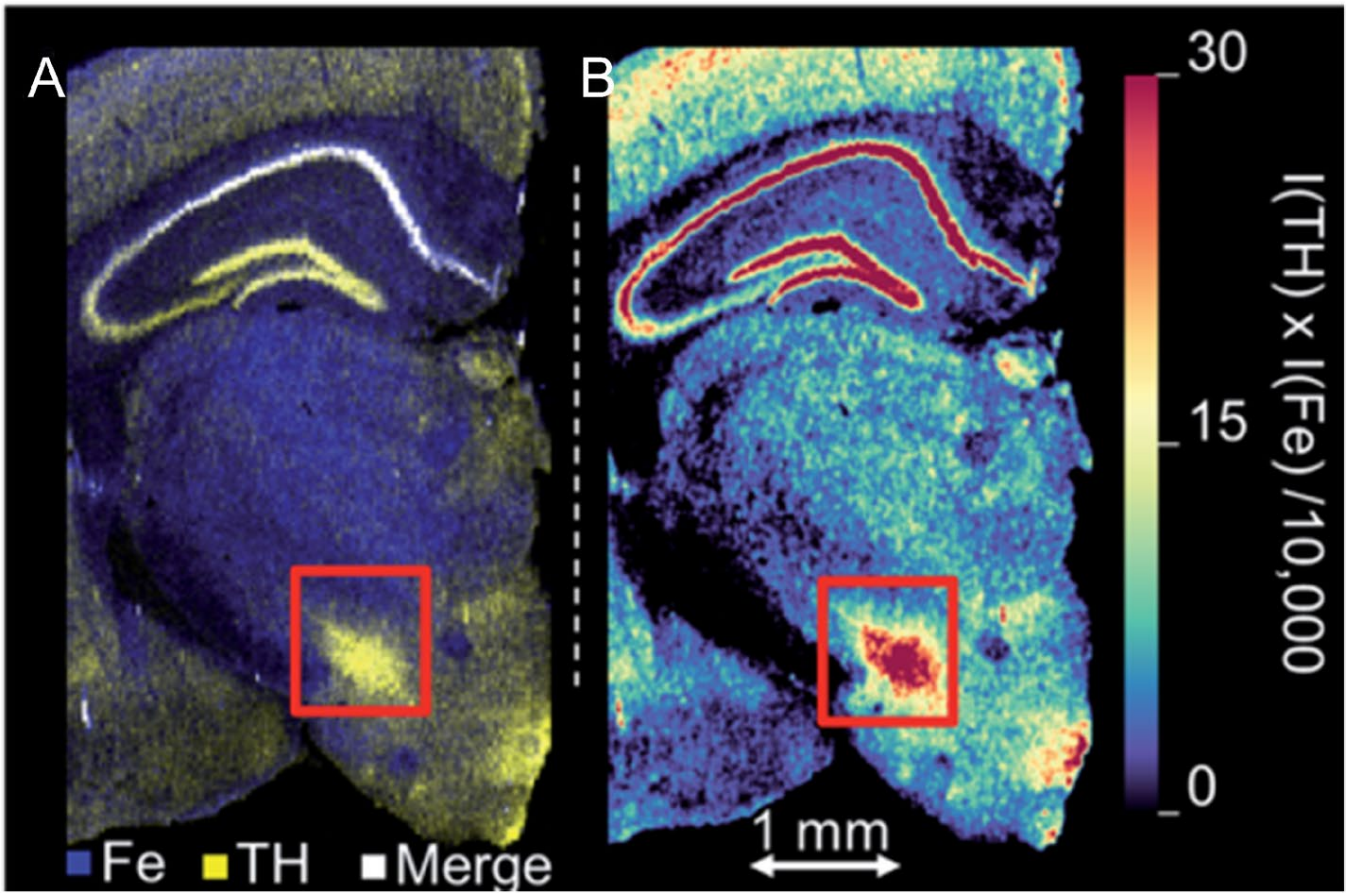
**A** An overlay of the Fe (blue) and tyrosine hydroxylase (TH) (yellow) distributions. **B** The Fe–TH score as a product of both intensities indicating areas of correlation. The red boxes indicate the area of the substantia nigra. Reprinted from Clases et al. [116] with permission from the Royal Society of Chemistry

Multi-omics approaches are becoming increasingly pressing to understand processes that occur at the metallomic/proteomic interface. For example, the simultaneous analysis of both proteins and elements is necessary to understand their complex biochemical interactions and IHC-assisted LA-ICP-MS may be employed to image both. The joint analysis of both elements and proteins requires consideration of factors that may affect native element distributions during sample preparation as processes like tissue fixation, embedding, washing, and staining may have a significant impact. Paul et al., Hare et al., and Clases et al. demonstrated the joint analysis of elements next to the protein tyrosine hydroxylase in murine brain sections [114–116]. Tyrosine hydroxylase was analysed as a dopamine proxy to indicate brain areas which show increased co-localisation with Fe (Figure 7). Co-localised dopamine and Fe may increase the levels of oxidative stress and could be interrogated as a risk index for parkinsonian neurodegeneration. Other reports on the joint analysis of proteins and elements were presented by Cruz-Alonso et al., who imaged the distribution of metallothionein in human ocular tissue using an Au NP label and compared its distribution with Cu and Zn [117]. In a later study, the authors demonstrated the imaging of Fe and ferroportin in the hippocampus of human brain tissue with Alzheimer’s disease [118]. Gonzalez de Vega et al. analysed MMP-11 as a novel metastatic biomarker in breast cancer. Following initial studies applying LA-ICP-MS and MALDI-MSI for the analysis of MMP-11 and its metal co-factor [119], the authors employed Au NPs labels for antibodies for IHC-assisted LA-ICP-MS and were able to compare the distribution of MMP-11 across tissues from control, cancer, and metastatic cancer patients [120]. In a subsequent study, Johnson et al. improved the approach further by employing lanthanide-based polymer tags and monoclonal antibodies for the multiplexed analysis of MMP-11 and CD45. They further applied SEC-ICP-MS and a signal thresholding algorithm to mask irrelevant image features and to determine the labelling degree of antibodies for quantitative comparisons [121].

**Fig. 8:**
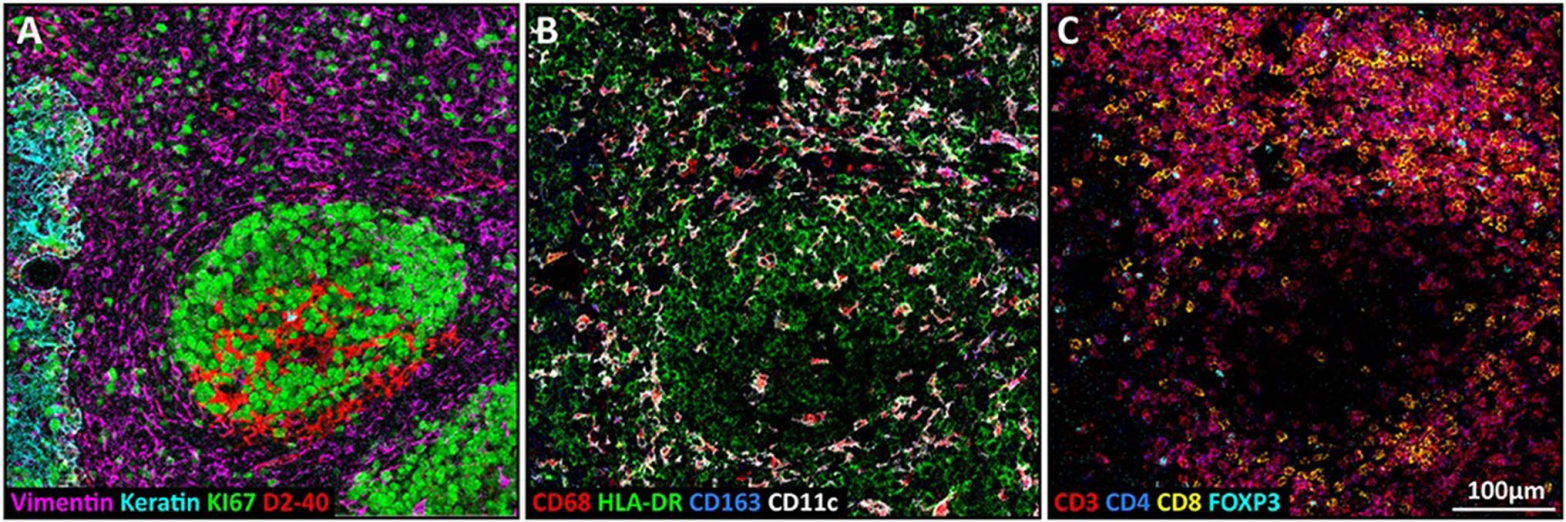
Imaging mass cytometry was used to detect structural (left), myeloid (middle), and lymphoid markers (right) in tonsil cancer tissues. Reprinted from Frontiers in Immunology, 10, 2019, 2534, M.E. Ijsselsteijn, R. van der Breggen, A.F. Sarasqueta, F. Koning, N.F.C.C. de Miranda, A 40-Marker Panel for High Dimensional Characterization of Cancer Immune Microenvironments by Imaging Mass Cytometry [102]. Copyright (2019), Ijsselsteijn et al.

The first highly multiplexed analysis was reported in 2014 by Giesen et al. targeting 32 different entities simultaneously [122]. Meanwhile, higher multiplexing degrees were achieved to investigate the microenvironment of tumour cells as demonstrated by Ijsselsteijn et al. and shown in Figure 8. The authors employed a 40-member antibody panel to image structural, myeloid, and lymphoid markers and to characterise cancer-immune cell interactions [102]. The application of large antibody panels in conjunction with high-resolution LA-ICP-MS (approx. 1 µm) allows the differentiation of cells and the study of their microenvironment, which can be used for cytometry approaches for cell profiling and consequently for the identification of rare subsets. Known as imaging mass cytometry (IMC), this technique advances previous single event approaches by accessing intra- and intercellular molecules to describe cellular metabolism, signalling, and the biochemical environment. While this offers a vast potential to study pathologies, it is also useful to differentiate healthy cell structures to depict and understand the biological traits for the generation of baseline data [123].

**Fig. 9:**
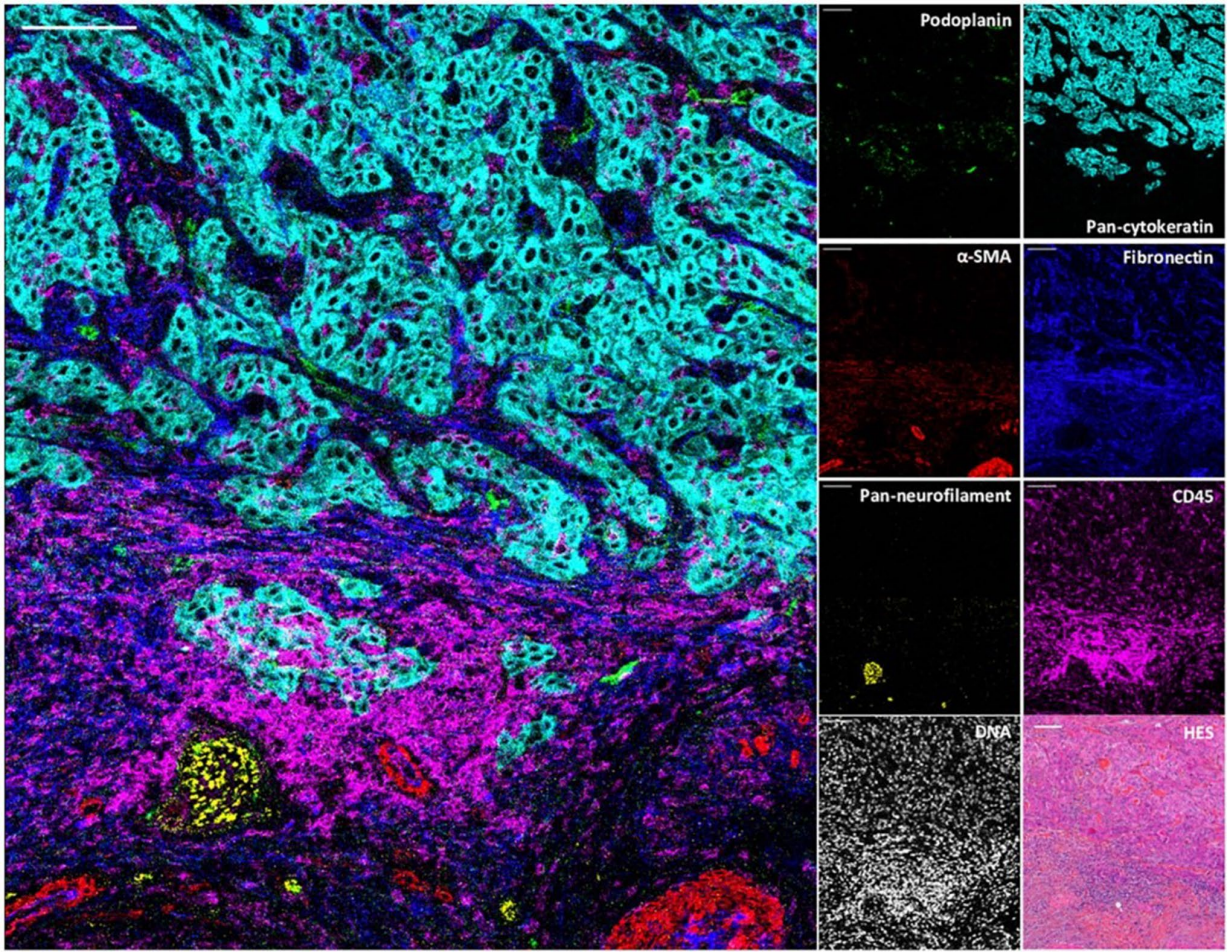
Visualisation by IMC of structural and cellular components in the tumour immune microenvironment of a cutaneous squamous cell carcinoma. Overlaid and single antibody signals representing lymphatic vessels (podoplanin), blood vessels and CAFs (α-SMA), nerve fibres (pan-neurofilament), tumour cells (pan-cytokeratin), ECM (fibronectin), and immune cells (CD45) are compared to nuclei and HES staining of the same region. Reprinted from Frontiers in Immunology, 12, 2021, 666233, R. Elaldi, P. Hemon, L. Petti, E. Cosson, B. Desrues, A. Sudaka, G. Poissonnet, E. Van Obberghen-Schilling, J-O. Pers, V.M. Braud, F. Anjuere, A. Meghraoui-Kheddar, High dimensional imaging mass cytometry panel to visualize the tumor immune microenvironment contexture [124]. Copyright (2021), Elaldi et al.

A wide range of applications of IMC have been suggested and especially the analysis of malignant diseases appears intriguing. The classification of the tumour immune microenvironment has the potential to provide novel perspectives on host responses, for selection of adequate immunotherapy, and to identify prognostic biomarkers [124]. Furthermore, cancer cells may be described with increasing detail providing new avenues for tumour differentiation that can be aligned with cancer genomics and current histopathology. An example for the imaging of structural and cellular components in the microenvironment of a tumour is shown in Figure 9. Additionally, aberrant signalling pathways may be investigated via phospho-specific antibodies as well as via surface growth factor receptors. All this endorses IMC as a clinical platform to study cancer progression and for developing new treatments [125, 126]. While the application for malignant diseases has a vast potential, capabilities of IMC go beyond the field of oncology and have a similar impact in other diseases such as autoimmune disorders and diabetes research [127, 128]. The potential role of IMC is versatile and promotes the refinement of diagnostic categories towards individualised treatment selection. Furthermore, considering the current costs for treatment schemes (e.g., cancer), clinical implementation of IMC may provide several advantages regarding expenditures alongside with patient care [125].

## Nano-scaled bioassays for ICP-MS

While medical imaging techniques remain the gold standard for diagnostic purposes, they are not suited for population-wide screening due to the required infrastructure, medical expertise, and high costs. Therefore, developments for screening strategies to pinpoint and characterise pathologies via minimal invasive and affordable techniques with high selectivity and sensitivity are in high demand [129]. The onset and progression of all pathologies are linked to aberrant changes on a molecular level, which actively remodel the biochemical environment long before adverse health effects and clinical manifestations are recognisable. The analysis of biomarkers, which are directly or indirectly associated with these changes, holds potential to improve diagnostics [130]. Cancer is one example for which early detection and intervention have a tremendous impact on treatment success and survival rate. The survival rate is strongly dependent on the cancer type, access to medical infrastructure, and progression at first detection [131]. Given the occurrence of cancer (approx. 19.3 million annual cases worldwide), substantial economic and research efforts are undertaken to establish a sustainable infrastructure to limit dissemination [132]. This sets a spotlight on minimal invasive techniques, which provide data that may be used to not only diagnose but also further characterise malignancies. In the case of cancer, circulating tumour-associated antigens (TAAs) are promising as easily accessible entities. Various TAAs have been identified in distinct cancer types and their combination and concentration are associated with the type of cancer, its cell proliferation, invasion, aggressiveness, and immune regulation [133]. Besides TAAs, autoantibodies have been identified as promising biomarkers which enable detection and classification of cancer years before clinical manifestations [134, 135]. Established methods for the identification and screening of biomarkers include serological analysis by recombinant cDNA expression cloning (SEREX). As a first stepping stone, this technique was soon complemented by protein microarrays, enzyme-linked immunoassays (ELISAs) as well as serological proteome analysis (SERPA) and multiple affinity protein profiling (MAPPing) [136]. With the latter two, advanced analytical instrumentation using separation techniques and high-resolution MS were included into the method portfolio for biomarker analysis.

A promising development in biomarker analysis exploits recent advances in the field of nanotechnology, immunochemistry, and elemental mass spectrometry. The possibility to label high affinity probes like nucleotides or antibodies with nanomaterials increases the labelling degree and consequently sensitivity so far that countable numbers of antibodies and their targets are detectable via ICP-MS [137, 138]. This extreme sensitivity is best exploited in a single event modality where pulses from single nanomaterials can be used as proxies for conjugated antibodies and their targets. The mechanism behind this approach is based on nano-scaled bioassays, where the surface of nanomaterials is functionalised with antibodies and used for heterogeneous or homogeneous immunoassays. Initial studies compared the capabilities of ICP-MS-based immunoassays, and limits of analysis could be improved substantially compared to ELISA [139–141]. Furthermore, possibilities for multiplexing are integrable [142, 143], suggesting the application of ICP-ToF-MS for simultaneous detection of different reporter particles in a single event segment. Functionalised magnetic beads have been demonstrated as versatile agents to improve sample preparation and limits of analysis via preconcentration while facilitating analyses of complex matrices including whole blood and serum [137, 138, 144]. Different mechanisms, other immunochemical techniques, and alternative affinity probes are available next to multiple-channel isotopic immunolabels to determine biomarkers. For example, particle aggregation [145] and dissolution [146] can be stimulated in the presence of specific analytes and used for calibration. Different strategies and applications as well as their merits will be reviewed in the following section.

### Protein-based assays

**Fig. 10:**
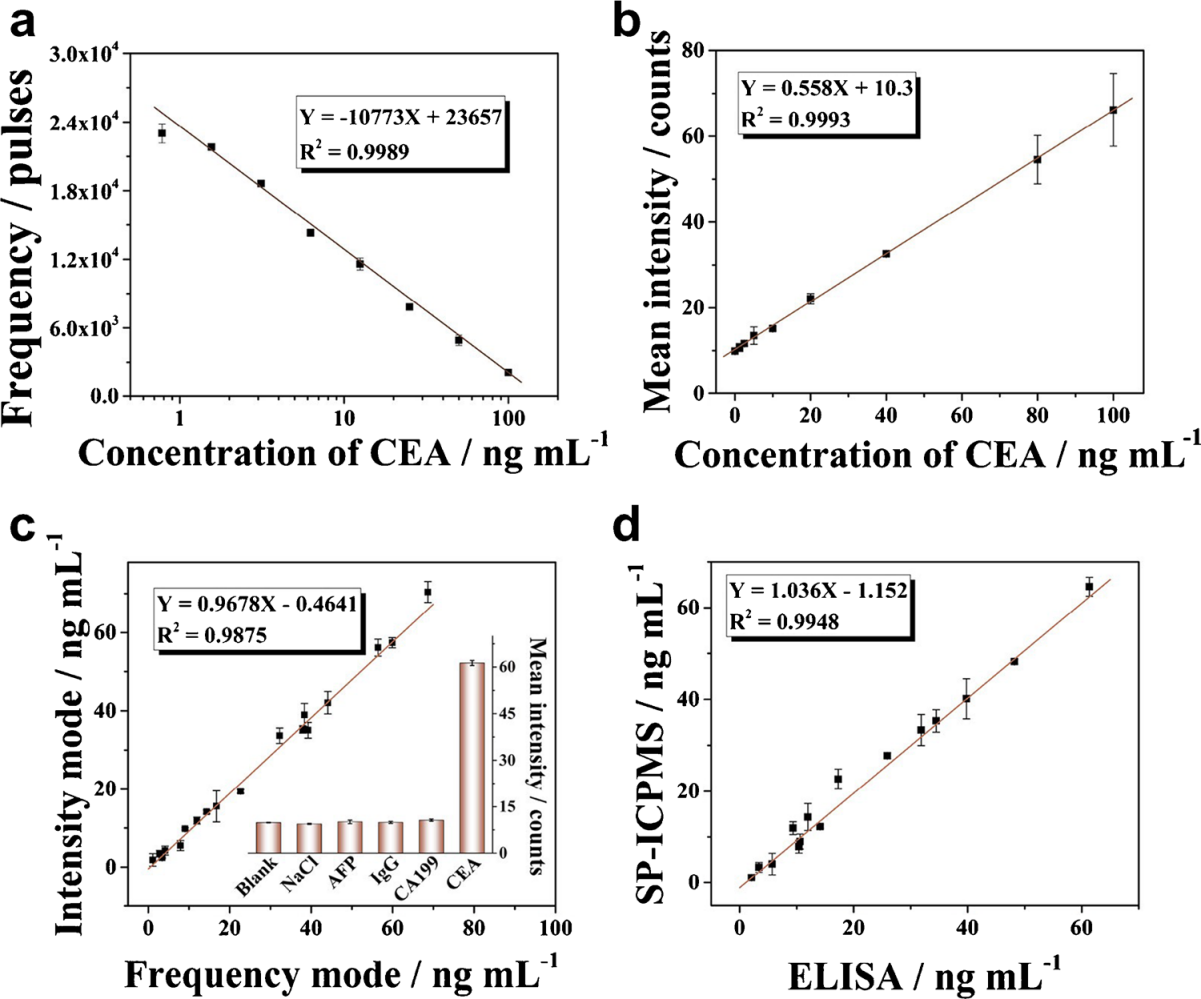
Antibody functionalised NPs were used in a homogeneous assay for SP ICP-MS. In the presence of CEA, aggregation was stimulated. The degree of aggregation can be expressed by the decreasing NPs number concentration (**a**) and the increasing signal intensity (**b**). (**c**) Correlation between both calibration modes. (**d**) Validation with an ELISA method. Reprinted with permission from Z. Huang, C. Wang, R. Liu, Y. Su. Y. Lv, Self-validated homogeneous immunoassay by single nanoparticle in-depth scrutinization, Analytical Chemsitry 92, 2020, 3, 2876–2881 [150]. Copyright (2020), American Chemical Society

In 2002, Baranov et al. reported an immunoassay which employed ICP-MS to analyse element tags and found limits of analysis for AFP, hCG, and Estriol ranging between 0.1 and 0.5 ng/mL and a linear response spanning across 3 orders of magnitude [147]. Zhang et al. followed up with a study immobilising monoclonal antibodies on microtiter plates to capture free AFP and hCG in serum [148]. The authors used Eu^3+^ and Sm^3+^ labelled antibodies for a sandwich immunoassay and, following dissociation, the rare earth metal concentrations were calibrated. Hu et al. suggested to employ SP ICP-MS for the highly sensitive detection in immunoassays [149]. They compared their method against the previous two studies and found that SP acquisition was improving limits of detection more than tenfold. Since these first studies, several other bioassay ICP-MS approaches have been reported. A relatively recent trend is the employment of functionalised nanomaterials which are bridged together by the biomolecule of interest leading to increasing particle aggregation. This homogeneous assay can be characterised by SP ICP-MS via recognising decreasing particle number concentrations and increasing pulse signal intensity (compare Figure 10). For example, Jiang et al. analysed CA19-9 as pancreatic cancer biomarker using two different antibodies which crosslinked Au NP in the presence of the biomarker [145]. Similarly, Huang et al. used an approach to analyse CEA via Au NP aggregation [150] and later expanded the method by inclusion of further metallic NPs (Ag, Pt, Au) to achieve a multiplexed analysis of CEA, CA125, and CA19-9 as pancreatic cancer biomarkers [151].

**Fig. 11:**
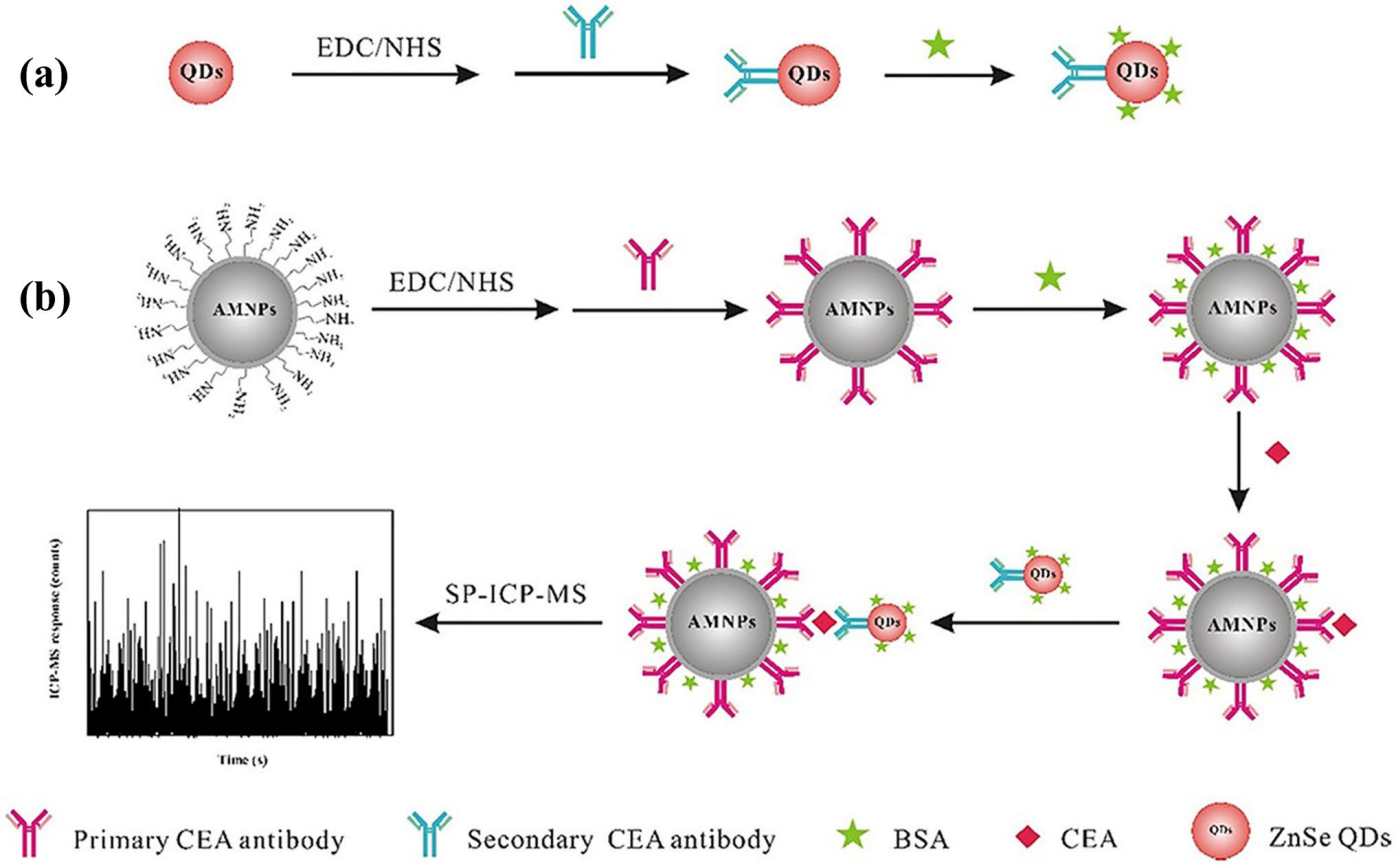
A magnetic immunoassay was developed for the analysis of CEA in human serum. QDs (**a**) and magnetic NPs (**b**) were functionalised with CEA specific antibodies. Following a blocking step to avoid unspecific binding, a sandwich immunocomplex was formed in serum, which could be extracted and analysed with SP ICP-MS monitoring the Zn signal. Reprinted from Analytica Chimica Acta, 1028, Y. Cao, G. Mo, J. Feng, X. He, L. Tang, C. Yu, B. Deng, Based on ZnSe quantum dots labeling and single particle mode ICP-MS coupled with sandwich magnetic immunoassay for the detection of carcinoembryonic antigen in human serum, 22–31 [137]. Copyright (2018), with permission from Elsevier

Peng et al. reported the analysis of CEA in human serum using a magnetic immunoassay. They used Hg as antibody label to build an immunocomplex which could be analysed via ICP-MS following magnetic extraction [141]. Zhang et al. used a similar approach to determine CEA and AFP. Using Au and Ag NPs as immunolabels, a magnetic sandwich assay was extracted, digested, and analysed for multiplexed calibration [138]. Cao et al. developed a magnetic immunoassay for the analysis of CEA in human serum employing QDs as immunolabels as shown in Figure 11. Following the construction of the immunocomplex and magnetic extraction, Zn could be analysed as proxy for CEA. The authors performed SP ICP-MS for the detection of multiple QDs immobilised on a single magnetic particle [137].

Zhang et al. suggested a sensitive ICP-MS and photothermal dual-readout assay as a platform for ultrasensitive point-of-care detection of exosomes [146]. Exosomes of pancreatic cancer cells expressing glypican-1 were targeted in a magnetic sandwich immunoassay incorporating Fe_3_O_4_@MnO_2_ nanoflowers. Using a dedicated enzymatic reaction, Mn^2+^ was stripped off the nanoflowers and quantified via ICP-MS as a proxy for exosome concentration. The nanoflower was subsequently analysed in a photothermal assay which has promise for exosome-based preliminary screenings.

### Nucleotide-based assays

The development of highly sensitive and rapid methods for detecting nucleic acids is of critical importance in biomedical studies. While current methods are mainly based on Southern Blotting, DNA microarrays, and PCR, ICP-MS-based bioassays offer new dedicated avenues for DNA detection. Oligo- and polynucleotides are increasingly utilised in bioassays for the analysis of biomolecules. In the form of aptamers, they can be employed similarly as antibodies and equipped with labels for ICP-MS detection. For example, Xing et al. employed an aptamer-based assay to detect thrombin [152]. Here, Au NPs were functionalised with aptamers and adsorbed on graphene oxide. In the presence of thrombin, Au NPs were desorbed due to a confirmation change of the aptamer and could be analysed by SP ICP-MS. Another application of aptamers was presented for the detection of exosomal proteins [153]. Y-, Eu-, and Tb-doped UCNPs were functionalised with the aptamer and immobilised on Au NPs. In the presence of exosomes with HER-2, EpCAM, and/or CD63, UCNPs were released and were analysed via ICP-MS. The authors further used linear discriminant analysis to differentiate exosomes from different cell lines which may be useful for further diagnostic insights.

**Fig. 12:**
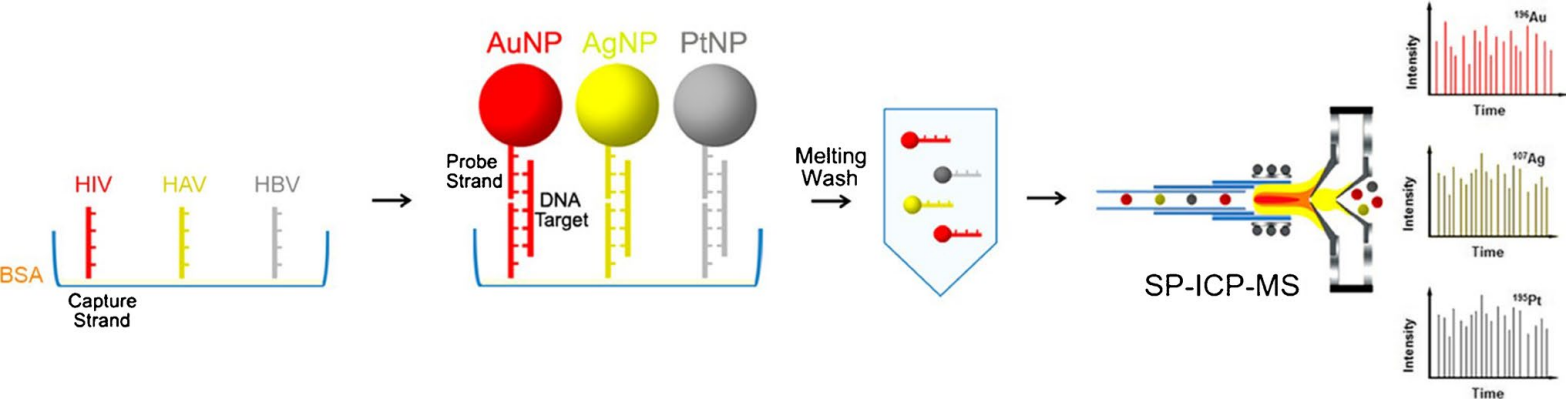
A heterogeneous bioassay for the detection of nucleic acids of HIV, HAV, and HBV was developed. The target DNA hybridised with both the immobilised capture strand and the metallic NPs. Above the DNA melting temperature, NPs were released and analysed via SP ICP-MS. Reprinted with permission from S. Zhang, G. Han, Z. Xing, S. Zhang, X. Zhang, Multiplex DNA assay based on nanoparticle probes by single particle inductively coupled plasma mass spectrometry, Analytical Chemistry 86, 2014, 7, 3541–3547 [143]. Copyright (2014), American Chemical Society

Compared to their application as aptamers, labelled nucleotide sequences were more frequently used as high affinity probes to detect specific DNA and RNA molecules. Han et al. developed a DNA hybridisation assay utilising the previously mentioned aggregation strategy. The aggregate degree and consequently the Au mass of aggregates were calibrated by SP ICP-MS and DNA levels as low as 1 pM could be determined [154]. Kang et al. immobilised three different DNA substrates carrying lanthanide tags on a magnetic bead [155]. In the presence of the target micro-RNA, three pairs of multicomponent nucleic acid enzymes were assembled where each pair was hybridised with the corresponding RNA cleaving the DNA substrate to release the lanthanide tag. Following magnetic separation, tags were analysed via ICP-MS as proxy for the targeted micro-RNA. Zhang et al. developed a heterogeneous multiplexed assay for SP ICP-MS to detect viral nucleic acids (HIV, HAV, HBV) as shown in Figure 12. They immobilised nucleotide strands on a 96-well plate, which were subsequently hybridised with the target DNA and functionalised with Pt, Ag, and Au NPs. Exceeding the melting temperature released the NPs for SP ICP-MS analysis [143]. Li et al. used target-induced hybridisation chain reaction to achieve controlled spherical nucleic acid assembly incorporating several Au NPs. The resulting Au NP aggregates were counted and calibrated by SP ICP-MS as a proxy for the DNA levels within a concentration range from 5 fM to 10 pM [156]. In a recent paper, Zhu et al. developed a triple cascade amplification strategy for the sensitive multiplexed analysis of cancer biomarkers via SP ICP-MS. A signal amplification module based on DNA-templated multiple metal nanoclusters were used to amplify SP signals. The advantages of this approach included high sensitivity, a wide dynamic range, and low background [157]. Luo et al. developed an ICP-MS-based multiplexed assay for detection of viral DNAs with lanthanide-coded oligonucleotide hybridisation and rolling circle amplification on bifunctional magnetic NPs. This ultrasensitive technique could determine concentrations as low as 90 zmol [158]. Zhang et al. presented an interesting approach for the detection of micro-RNA. Magnetic microparticles were linked to lanthanide tags via a bifunctional oligonucleotide. The target RNA could hybridise with the oligonucleotide to form a DNA/miRNA heteroduplex, which was subsequently cleaved via a duplex-specific nuclease. The released elemental tag was analysed by ICP-MS and the RNA was reused for repeated hybridisation and, consequently, amplification [159]. Recently, Xu et al. developed a homogeneous assay to detect the nucleic acids of SARS-CoV-2 and influenza A. They functionalised Au and Ag NPs that aggregated in the presence of the respective viral nucleic acid, which could be observed in SP ICP-MS via the NP count and signal intensity [160].

## Conclusion and future perspectives

The technological and methodological advances over the last years promoted ICP-MS as a versatile and powerful platform which enables much more than the sole determination of total elemental concentrations. Especially the maturation of mass analysers (particularly ToF analysers), hyphenated techniques, single event analysis, and immunochemistry-based protocols redefined traditional boundaries in ICP-MS and provided multifaceted perspectives on diverse and pressing medical questions. The advantages and opportunities of some facets have been recognised in the medical communities and clinical translation appears extremely rewarding. This includes facets like mass cytometry as well as imaging mass cytometry which evolved into accepted platform technologies for high-dimensional cell analysis. They may be used complementary to genomic methods and hold vast potential for personalised treatment selection while improving diagnostics, therapeutics, and disease prevention. Especially in the field of (immuno-)oncology and autoimmunity but also other fields, dozens of clinical research trials are under way and the momentum is increasing. As such, clinical translation seems realistic if not even imminent. The additional targeting of endogenous elements is a current research direction which may complement mass cytometry. Endogenous elements have been correlated with various pathologies and concurrent analysis of antibody-conjugated isotope tags and essential elements increases the biomarker panel size without major setbacks.

In conjunction with nanotechnology, these new facets of ICP-MS have further the capability to push the limits of biomarker analysis, to enable high sample throughput and consequently fast screening platforms, and to provide increased information depth to improve diagnostic and characterisation of pathologies. Diverse mechanisms for ICP-MS-based bioassays were developed and promise high sensitivity, rapid analysis, and straight forward application for the multiplexed calibration of protein and nucleic acid-based biomarkers for diagnostic and prognostic purposes. In this context, ICP-MS techniques may have the potential to address the demands for more affordable as well as minimal invasive large-scale screening strategies to detect the onset of malignancies and therefore to improve human health.

The harnessing of nanotechnology for new drug delivery and imaging approaches is another driving and innovative force in medicine. Candidates for nanomedicines have already been approved and an increasing number of new candidates are trialled. ICP-MS, its hyphenated techniques, and acquisition modes are capable to fill an important analytical gap to characterise and benchmark nanomedicines by addressing important questions raised in early and advanced preclinical stages. ICP-MS techniques provide elegant approaches to inquire biosafety, fate, efficacy, biochemical, and biophysical interactions of nanomaterials. Besides nanomedicines, these toxicological considerations become increasingly relevant when considering the constant and inevitable exposure to nano-scaled contaminants.

Despite the broad method portfolio of ICP-MS, complementary and multimodal approaches are required for more holistic interpretation and to better understand biochemical pathways. Several molecule selective approaches and high-resolution techniques such as for example MALDI-MSI [119, 161] and SIMS [162] in bioimaging, LC-ESI-HRMS [163] in speciation analysis or SERS [49] in particle analysis were demonstrated to provide additional important information. Also, established medical techniques such as CT or MRI were applied in conjunction with ICP-MS methods to construct novel models and attain new perspectives [164–166]. It can be expected that the development and application of multimodal approaches will increase in the future to provide new depths of information and to generate complementary insights.

ICP-MS techniques and applications are becoming increasingly interdisciplinary and can be operated at the interface of medicine, environmental sciences, nanotechnology, and biochemistry. Furthermore, the increasing complexity and size of data reaches levels where new software solutions as well as advanced statistical evaluations/models are inevitable. As such, data sciences are and will be increasingly integrated into bioanalytical workflows and information processing. While the capabilities of some facets are dawning on diverse research communities, the current potential of ICP-MS is by far not utilised and more joint explorations are required. This calls for an increased communication between groups working in the medical and natural sciences and their success will likely depend on close collaborations and complementary initiatives for a transdisciplinary approach.
